# Impact of early empirical antifungal therapy on prognosis of sepsis patients with positive yeast culture: A retrospective study from the MIMIC-IV database

**DOI:** 10.3389/fmicb.2022.1047889

**Published:** 2022-11-17

**Authors:** Zhi-ye Zou, Kai-jun Sun, Guang Fu, Jia-jia Huang, Zhen-jia Yang, Zhi-peng Zhou, Shao-lin Ma, Feng Zhu, Ming Wu

**Affiliations:** ^1^Department of Critical Care Medicine and Hospital Infection Prevention and Control, Shenzhen Second People’s Hospital & First Affiliated Hospital of Shenzhen University, Health Science Center, Shenzhen, China; ^2^Department of Cardiovascular Medicine, Weifang People's Hospital, Weifang, China; ^3^Department of Gastroenterology, The First Affiliated Hospital of University of South China, Hengyang, China; ^4^Shantou University Medical College, Shantou, China; ^5^Department of Critical Care Medicine, Shanghai East Hospital, Tongji University School of Medicine, Shanghai, China; ^6^Burn and Trauma ICU, The First Affiliated Hospital, Naval Medical University, Shanghai, China

**Keywords:** sepsis, yeast, outcomes, antifungal therapy, positive culture

## Abstract

**Background:**

Mortality and other clinical outcomes of culture-negative and culture-positive among patients with fungal sepsis have not been documented, and whether antifungal therapy prior to fungal culture reports is related to decreased mortality among patients remains largely controversial. This study aimed to determine the mortality and other clinical outcomes of patients with positive yeast cultures and further investigate the effects of initial empiric antifungal therapy.

**Methods:**

A retrospective study was conducted among septic patients using the Medical Information Mart for Intensive Care (MIMIC)-IV database. Patients with sepsis were divided into two groups based on first fungal culture status during intensive care unit (ICU) stay, and initial empirical antifungal therapy was prescribed based on physician’s experience prior to fungal culture reports within 48 h. The primary outcome was in-hospital all-cause mortality. The secondary outcomes were 30-day all-cause mortality, 60-day all-cause mortality, length of ICU stay and length of hospital stay. Multivariate logistic regression, propensity score matching (PSM), subgroup analyses and survival curve analyses were performed.

**Results:**

This study included 18,496 sepsis patients, of whom 3,477 (18.8%) had positive yeast cultures. Patients with positive yeast cultures had higher in-hospital all-cause mortality, 60-day all-cause mortality, and longer lengths of ICU stay and hospital stay than those with negative yeast cultures after PSM (all *p* < 0.01). Multivariate logistic regression analysis revealed that positive yeast culture was a risk factor for in-hospital mortality in the extended model. Subgroup analyses showed that the results were robust among the respiratory infection, urinary tract infection, gram-positive bacterial infection and bacteria-free culture subgroups. Interestingly, empiric antifungal therapy was not associated with lower in-hospital mortality among patients with positive yeast cultures, mainly manifested in stratification analysis, which showed that antifungal treatment did not improve outcomes in the bloodstream infection (odds ratio, *OR* 2.12, 95% *CI*: 1.16–3.91, *p* = 0.015) or urinary tract infection groups (*OR* 3.24, 95% *CI*: 1.48–7.11, *p* = 0.003).

**Conclusion:**

Culture positivity for yeast among sepsis patients was associated with worse clinical outcomes, and empiric antifungal therapy did not lower in-hospital all-cause mortality in the bloodstream infection or urinary tract infection groups in the ICU.

## Introduction

Sepsis is characterized by life-threatening organ dysfunction caused by a dysregulated host response to infection (Singer et al., 2016). Due to its high incidence and mortality, sepsis is considered one of the top three most costly diseases (Liang and Soni, 2020) and has been recognized as a global health priority by the World Health Organization, posing a substantial threat to human health (Reinhart et al., 2017). Many previous studies have proven that positive and negative bacterial cultures do not affect mortality among sepsis patients (Li et al., 2021). Although the most common pathogens that cause sepsis are bacteria, fungi account for a nonnegligible percentage of isolated pathogens in sepsis (Vincent et al., 2009, 2018). However, whether positive or negative fungal cultures are associated with mortality among sepsis patients has not been reported in the literature. Antimicrobial therapy is the primary approach for managing infections and improving the prognosis of patients with sepsis (Cecconi et al., 2018). Although prompt initiation of appropriate empiric antifungal treatment theoretically reduces mortality, there is disagreement between studies (Morrell et al., 2005; Garey et al., 2006; Marriott et al., 2009; Kollef et al., 2012). Recently, a meta-analysis showed that prophylactic therapies did not improve the prognosis of critically ill patients with fungal sepsis (Cortegiani et al., 2017). However, these available studies have not demonstrated a causal relationship between antifungal treatment and outcome, nor have they clarified the role of treatment timing.

Since yeast-like fungi account for more than 70% of fungal sepsis (Delaloye and Calandra, 2014) and there are few studies on the clinical characteristics and outcomes of critically ill patients with yeast-like fungi, in this study, we focused on the baseline characteristics and clinical outcomes of sepsis patients with different yeast culture results. We then described risk factors for higher in-hospital mortality among patients with positive yeast cultures and further analyzed the impact of early empirical antifungal therapy on outcomes among these patients.

## Materials and methods

### Study design and data source

We performed a retrospective study using Medical Information Mart for Intensive Care IV (MIMIC-IV) version 1.0 data (https://physionet.org/content/mimiciv/1.0/), which includes the medical information of ICU patients in a Boston hospital in the United States over a 10-year study period. The establishment of the database was approved by the Massachusetts Institute of Technology (Cambridge, MA) and the Institutional Review Boards of Beth Israel Deaconess Medical Center (Boston, MA). Data were extracted from the MIMIC-IV database using Structured Query Language (SQL) with Navicat Premium (version 12.0.28) and consisted of age, sex, race, insurance, weight, source of admission, medical history, Sequential Organ Failure Assessment (SOFA) score, vital signs, laboratory outcomes, infection sites, gram-positive bacterial infection, gram-negative bacterial infection, in-hospital management, antifungal agents, microbial culture results and survival data. One author (Zhi-Ye Zou) obtained access to this database (certification number 35951237) and was responsible for data extraction.

### Participants

All patients who met the Third International Consensus Definitions for Sepsis and Septic Shock (Sepsis-3) criteria and had a lactate level ≥2 mmol/l were included. The exclusion criteria were as follows: age <18 or age >90 years; length of ICU stay <24 h; fungal negativity within 48 h but fungal positivity after 48 h; or *Aspergillus* or *Cryptococcus* positivity. Only the first admission was considered for patients with two or more ICU admissions.

#### Research procedures and definitions

In this study, “sepsis” was defined as a documented or suspected infection and a SOFA score ≥2 (Singer et al., 2016). An early positive culture was defined as at least one positive result from multiple specimens obtained from the patient within 48 h before and after admission to the ICU. The time for yeast culture results was 3.44 days [median (IQR), 3.44 (1.73, 6.32)] after specimen collection. The infection site was determined by International Classification of Diseases, 9^th^ Revision (ICD-9) or ICD-10 codes provided in the MIMIC-IV database (icd10_code or icd9_code). If there was no corresponding ICD code, “other site of infection” was assigned. The bacteria-free group was defined as patients for whom no bacteria were cultured but included those for whom fungi were or were not cultured. In-hospital management, including renal replacement therapy, the use of vasopressor drugs, and mechanical ventilation, referred to the use of related treatment methods within 24 h of admission to the ICU. The maximum SOFA score, vital signs, and laboratory outcomes referred to the results obtained within the first 24 h of ICU admission. Early antifungal agents referred to the use of antifungal drugs within 48 h before and after ICU admission. Antifungal drugs included azole antifungals (fluconazole and voriconazole), echinocandins (caspofungin and micafungin), and amphotericin B. The routes of fungal administration included oral, intravenous, and aerosol administration. Later, antifungal agent use referred to the use of the above antifungal drugs only after admission to the ICU for 48 h.

#### Exposure and outcomes

The patients were divided into two groups according to the final culture status of the specimens, which were collected within 48 h before and after ICU admission. We divided patients into a negative yeast culture group and positive yeast culture group for further analysis. The primary outcome was in-hospital all-cause mortality. Secondary outcomes were 30-day all-cause mortality, 60-day all-cause mortality, length of ICU stay, and length of hospital stay.

### Statistical analysis

Missing data were processed by multiple imputation (Supplementary Table S1). Data are presented as the means ± standard deviations (means ± SDs) or percentages (*n*%) as appropriate. Differences between groups were analyzed using Student’s *t*-test and the *χ*^2^ test, as appropriate.

Propensity score matching (PSM) was performed to balance confounding factors. Variables, including all variables in Table 1, were chosen to generate the PS based on clinical significance and previous studies. We constructed a multivariable logistic regression model. A one-to-one nearest neighbor matching algorithm was applied using a caliper width of 0.2. After PSM, we used standardized mean differences (SMDs) and *p* values to judge the balance of baseline characteristics between the groups. When the SMD of a variable was larger than 0.1, an imbalance between groups was considered. Finally, 3,163 patients in each group were well matched, and their data were extracted for further analysis. Survival analysis was conducted using Kaplan–Meier and log-rank tests before and after PSM. Additionally, we calculated the in-hospital mortality absolute risk reduction based on fungal culture status.

**Table 1:** Baseline demographics and clinical characteristics of the patients with negative yeast cultures and positive yeast cultures.

	All patients (*n* = 18,496)	Propensity score matching						
		Before			After			
		Negative yeast cultures (*n* = 15,019)	Positive yeast cultures (*n* = 3,477)	*p-*value	Negative yeast cultures (*n* = 3,163)	Positive yeast cultures (*n* = 3,163)	*p-*value	SMD
Baseline characteristics								
Age (year), mean (*SD*)	65.24 (15.41)	65.46 (15.40)	64.25 (15.38)	<0.001	65.00 (15.69)	64.62 (15.39)	0.34	0.024
Male, *n* (%)	10,850 (58.7)	9,064 (60.4)	1786 (51.4)	<0.001	1,627 (51.4)	1,653 (52.3)	0.51	0.016
White, *n* (%)	12,239 (66.2)	9,995 (66.5)	2,244 (64.5)	0.024	2020 (63.9)	2043 (64.6)	0.55	0.015
Insurance, Medicare, *n* (%)	8,708 (47.1)	7,058 (47.0)	1,650 (47.5)	0.62	1,539 (48.7)	1,515 (47.9)	0.55	0.015
Weight (kg), mean (*SD*)	82.17 (24.79)	82.09 (24.84)	82.50 (24.58)	0.38	81.97 (24.66)	82.01 (24.09)	0.95	0.001
Admission (emergency), *n* (%)	9,199 (49.7)	7,400 (49.3)	1799 (51.7)	0.009	1,665 (52.6)	1,645 (52.0)	0.61	0.013
History of disease, *n* (%)								
Congestive heart failure	5,827 (31.5)	4,599 (30.6)	1,228 (35.3)	<0.001	1,161 (36.7)	1,121 (35.4)	0.29	0.026
Peripheral vascular disease	2,375 (12.8)	1898 (12.6)	477 (13.7)	0.086	433 (13.7)	433 (13.7)	1.00	<0.001
Chronic pulmonary disease	4,838 (26.2)	3,731 (24.8)	1,107 (31.8)	<0.001	989 (31.3)	996 (31.5)	0.85	0.005
Renal disease	4,520 (24.4)	3,623 (24.1)	897 (25.8)	0.038	825 (26.1)	808 (25.5)	0.63	0.012
Rheumatic disease	725 (3.9)	562 (3.7)	163 (4.7)	0.010	143 (4.5)	139 (4.4)	0.81	0.006
Diabetes without cc	4,792 (25.9)	3,798 (25.3)	994 (28.6)	<0.001	960 (30.4)	897 (28.4)	0.082	0.044
Diabetes with cc	2079 (11.2)	1,658 (11.0)	421 (12.1)	0.072	405 (12.8)	388 (12.3)	0.52	0.016
Metastatic solid tumor	1,212 (6.6)	971 (6.5)	241 (6.9)	0.32	218 (6.9)	225 (7.1)	0.73	0.009
Severe liver disease	1806 (9.8)	1,269 (8.4)	537 (15.4)	<0.001	415 (13.1)	439 (13.9)	0.38	0.022
Malignant cancer	2,656 (14.4)	2,103 (14.0)	553 (15.9)	0.004	510 (16.1)	512 (16.2)	0.95	0.002
AIDS	160 (0.9)	120 (0.8)	40 (1.2)	0.044	30 (0.9)	36 (1.1)	0.46	0.019
Maximum SOFA score on 1^st^ day, mean (*SD*)	6.89 (3.95)	6.40 (3.66)	9.01 (4.43)	<0.001	8.53 (4.34)	8.60 (4.26)	0.48	0.018
Vital signs at 1^st^ day, mean (*SD*)								
MAP (mmHg)	76.39 (10.24)	76.63 (10.26)	75.35 (10.09)	<0.001	75.43 (10.15)	75.60 (10.20)	0.52	0.016
Maximum heart rate (bpm)	107.55 (21.18)	106.43 (20.90)	112.34 (21.68)	<0.001	112.23 (22.25)	111.78 (21.52)	0.41	0.021
Maximum respiratory rate (bpm)	28.77 (6.53)	28.50 (6.42)	29.84 (6.87)	<0.001	29.80 (6.90)	29.69 (6.80)	0.50	0.017
Maximum temperature (°C)	37.52 (0.80)	37.50 (0.78)	37.57 (0.87)	<0.001	37.56 (0.86)	37.56 (0.87)	0.89	0.003
Laboratory outcomes, mean (*SD*)								
Minimum white blood cell (10^9^/L)	10.72 (6.19)	10.50 (5.97)	11.66 (7.01)	<0.001	11.59 (7.02)	11.59 (6.86)	1.00	<0.001
Maximum white blood cell (10^9^/L)	15.58 (8.77)	15.23 (8.51)	17.06 (9.64)	<0.001	16.75 (9.63)	16.90 (9.50)	0.54	0.015
Platelets min	174.26 (102.69)	172.63 (98.98)	181.30 (117.14)	<0.001	185.46 (118.87)	183.17 (115.49)	0.44	0.020
Infection sites, *n* (%)								
Respiratory infection	6,671 (36.1)	4,536 (30.2)	2,135 (61.4)	<0.001	1901 (60.1)	1860 (58.8)	0.29	0.026
Urinary tract infection	3,384 (18.3)	2,608 (17.4)	776 (22.3)	<0.001	691 (21.8)	696 (22.0)	0.88	0.004
bloodstream infection	1,630 (8.8)	1,041 (6.9)	589 (16.9)	<0.001	466 (14.7)	460 (14.5)	0.83	0.005
Abdominal infection	1,208 (6.5)	790 (5.3)	418 (12.0)	<0.001	303 (9.6)	325 (10.3)	0.35	0.023
Central nervous infection	236 (1.3)	167 (1.1)	69 (2.0)	<0.001	57 (1.8)	62 (2.0)	0.64	0.012
Other sites infection	8,397 (45.4)	7,660 (51.0)	737 (21.2)	<0.001	694 (21.9)	729 (23.0)	0.29	0.027
Gram-positive bacteria, *n* (%)	3,159 (17.1)	2,334 (15.5)	825 (23.7)	<0.001	743 (23.5)	729 (23.0)	0.68	0.010
Gram-negative bacteria, *n* (%)	2,480 (13.4)	1780 (11.9)	700 (20.1)	<0.001	649 (20.5)	618 (19.5)	0.33	0.024
In-hospital management, *n* (%)								
Renal replacement therapy	1,305 (7.1)	948 (6.3)	357 (10.3)	<0.001	283 (8.9)	294 (9.3)	0.63	0.012
Vasopressor use	9,401 (50.8)	7,143 (47.6)	2,258 (64.9)	<0.001	1917 (60.6)	1967 (62.2)	0.20	0.032
Mechanical ventilation	8,551 (46.2)	6,444 (42.9)	2,107 (60.6)	<0.001	1852 (58.6)	1842 (58.2)	0.80	0.006
Early Antifungal agent, *n* (%)	663 (3.6)	418 (2.8)	245 (7.0)	<0.001	197 (6.2)	195 (6.2)	0.92	0.003
Azole antifungals	412 (2.2)	274 (1.8)	140 (4.0)	<0.001	123 (3.9)	115 (3.6)	0.60	0.013
Echinocandin	248 (1.3)	148 (1.0)	100 (2.9)	<0.001	76 (2.4)	79 (2.5)	0.81	0.006
Amphotericin	37 (0.2)	24 (0.2)	13 (0.4)	0.011	14 (0.4)	9 (0.3)	0.30	0.026
Later antifungal agent, *n* (%)	657 (3.6)	219 (1.5)	438 (12.6)	<0.001	190 (6.0)	229 (7.2)	0.049	0.050

SMD, standardized mean difference; SD, standard deviation; CC, chronic complication; AIDS, Acquired Immune Deficiency Syndrome; SOFA, Sequential Organ Failure Assessment; MAP, mean blood pressure; bpm, beats per minute or breaths per minute.

An extended logistic model was applied to adjust for covariates that might affect outcomes. Stratified analyses were conducted to explore whether the culture status of early specimens and in-hospital mortality differed among the various subgroups classified by site of infection and microbial culture results.

Based on clinical experience, the effect of early antifungal treatment on in-hospital mortality among patients with different infection sites with or without evidence of fungal infection was analyzed. Univariate analyses were performed; then, variables were initially selected according to clinical experience. Variables with *p* < 0.1 were included in multivariate analysis to further analyze the relationship between early antifungal treatment and in-hospital mortality.

Most variables had no missing values, although a few variables had less than 6% missing data. Multiple imputation was used for missing values under the assumption that they were missing at random. Two-tailed p values less than 0.05 were considered statistically significant. All statistical analyses were performed using Stata 15.1 (StataCorp, College Station, TX, United States) and R 4.0.1 software for Windows.

## Results

### Baseline characteristics of sepsis patients with positive yeast cultures

**Figure 1:**
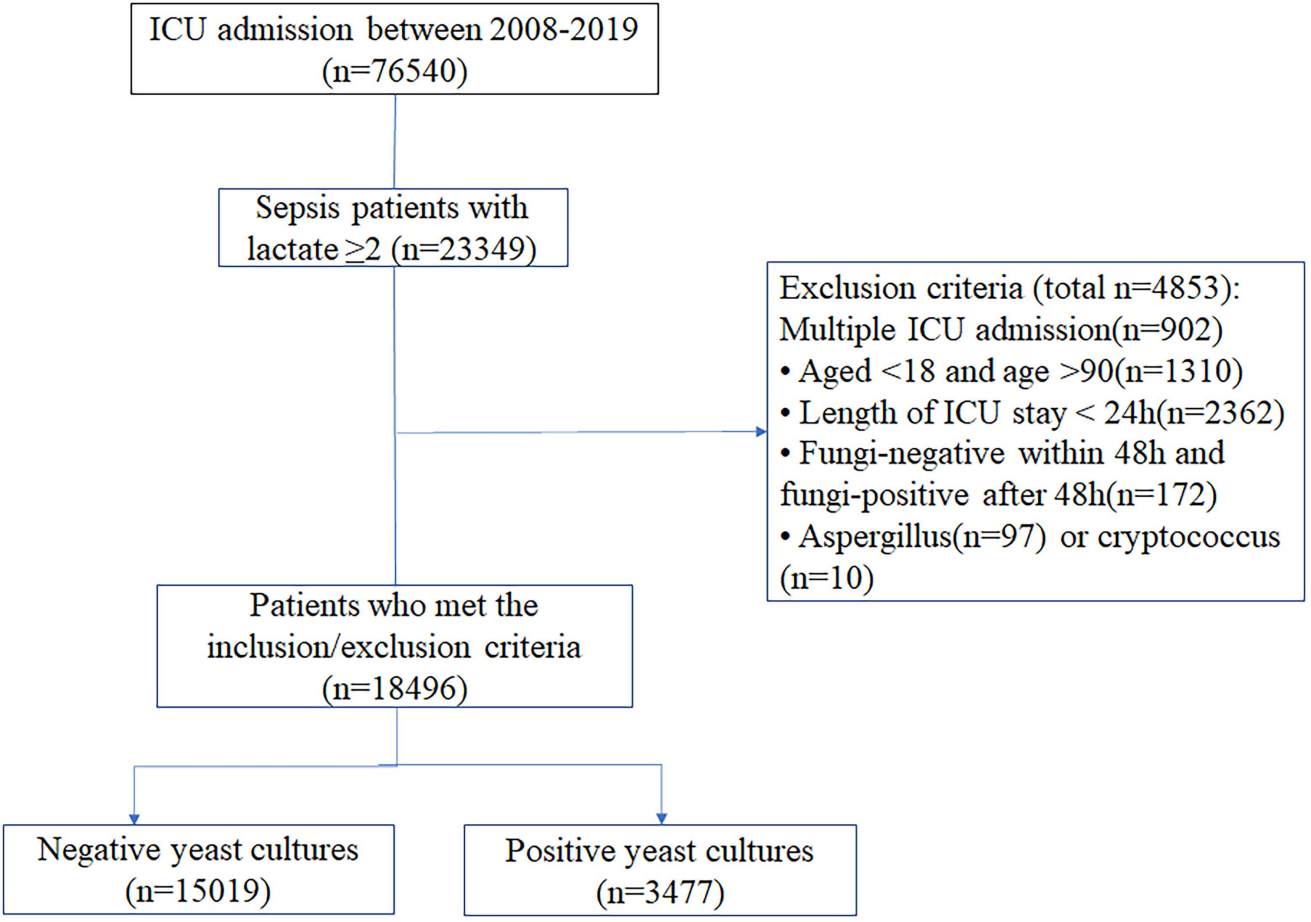
Flowchart of patient selection.

This study included 18,496 sepsis patients, of whom 3,477 (18.8%) had positive yeast cultures (Figure 1). Most of the data were available (Supplementary Table S1). The baseline characteristics of patients upon ICU admission are shown in Table 1. Compared with the yeast culture-negative group, the yeast culture-positive group was slightly younger and more likely to be female and nonwhite. The yeast culture-positive group also had more comorbidities, higher SOFA scores, and more sites of infection. As expected, patients with positive yeast cultures received more antifungal drugs, either early (7.0% vs. 2.8%, *p* < 0.01) or late (12.6% vs. 1.5%, *p* < 0.01) antifungal drugs.

### Clinical outcomes of sepsis patients with positive yeast cultures and subgroup analysis for infection site

Compared to patients with negative yeast cultures, patients with positive yeast cultures had higher in-hospital all-cause mortality (13.8% vs. 31.1%, *p* < 0.001), 30-day all-cause mortality (14.2% vs. 28.1%, *p* < 0.001), and 60-day all-cause mortality rates (15.3% vs. 32.0%, *p* < 0.001; Table 2). After PSM (Supplementary Figure S1), the hospital all-cause mortality, 30-day all-cause mortality and 60-day all-cause mortality of patients with positive yeast cultures increased by 3.1, 1.0, and 2.5%, respectively, compared with those of patients with negative yeast cultures. In addition, the positive yeast culture group had longer durations of ICU and hospital stays than the negative yeast culture group (Table 2). Kaplan–Meier curves visually showed the differences in outcomes between the two groups (Supplementary Figure S2A). To balance confounding factors, we used logistic extended model analysis and the PSM method. Extended model analysis showed that positive yeast culture was a risk factor for in-hospital mortality, with odds ratios (*OR*s) ranging from 1.32 to 2.91 (Table 3).

**Table 2:** Primary and Secondary outcomes before and after PSM.

Variables	All patients (*n* = 18,496)	Before PSM				After PSM			
		Negative yeast cultures (*n* = 15,019)	Positive yeast cultures (*n* = 3,477)	ARR (95%*CI*)	*p*-value	Negative yeast cultures (*n* = 3,163)	Positive yeast cultures (*n* = 3,163)	ARR (95%*CI*)	*p*-value
*Primary outcome*									
In-hospital mortality, *n* (%)	3,160 (17.1%)	2078 (13.8)	1,082 (31.1)	17.3 (15.6,18.9)	<0.001	828 (26.2)	926 (29.3)	3.1 (0.9–5.3)	0.006
*Secondary outcomes*									
30-day mortality, *n* (%)	3,102 (16.8%)	2,126 (14.2)	976 (28.1)	13.9 (12.3,15.5)	<0.001	826 (26.1)	857 (27.1)	1.0 (−0.3,1.0)	0.38
60-day mortality, *n* (%)	3,407 (18.4%)	2,294 (15.3)	1,113 (32.0)	16.7 (15.1,18.4)	<0.001	882 (27.9)	962 (30.4)	2.5 (0.3,4.8)	0.027
Length of ICU stay (days), mean (*SD*)	5.63 (6.53)	4.51 (4.91)	10.47 (9.69)	NA	<0.001	6.28 (6.44)	9.62 (8.59)	NA	<0.001
Length of hospital stay (days), mean (*SD*)	13.63 (14.99)	11.30 (11.27)	23.70 (22.85)	NA	<0.001	14.23 (16.55)	22.77 (22.23)	NA	<0.001

ARR, absolute risk reduction; CI, confidence interval; ICU, intensive care unit; SD, standard deviation; NA, Not application.

**Table 3:** Association between early positive yeast cultures and hospital mortality using an extended model approach.

	Odds ratio	95% Confidence interval	*p*
Model 1	2.81	(2.58–3.06)	<0.001
Model 2	2.91	(2.66–3.17)	<0.001
Model 3	2.75	(2.51–3.00)	<0.001
Model 4	1.63	(1.47–1.80)	<0.001
Model 5	1.40	(1.26–1.55)	<0.001
Model 6	1.32	(1.19–1.47)	<0.001

Model 1 = early positive yeast cultures. Model 2 = Model 1 + (age, gender, ethnicity, insurance, weight, admission). Model 3 = Model 2 + (History of disease including congestive heart failure, peripheral vascular disease, chronic pulmonary disease, renal disease, diabetes without cc, diabetes with cc, metastatic solid tumor, severe liver disease, malignant cancer, and Aids). Model 4 = Model 3 + sofa + (Vital signs at first day) + (Laboratory outcomes). Model 5 = Model 4 + (infection sites) + (In-hospital management). Model 6 = Model 5 + (Antifungal agent).

**Figure 2:**
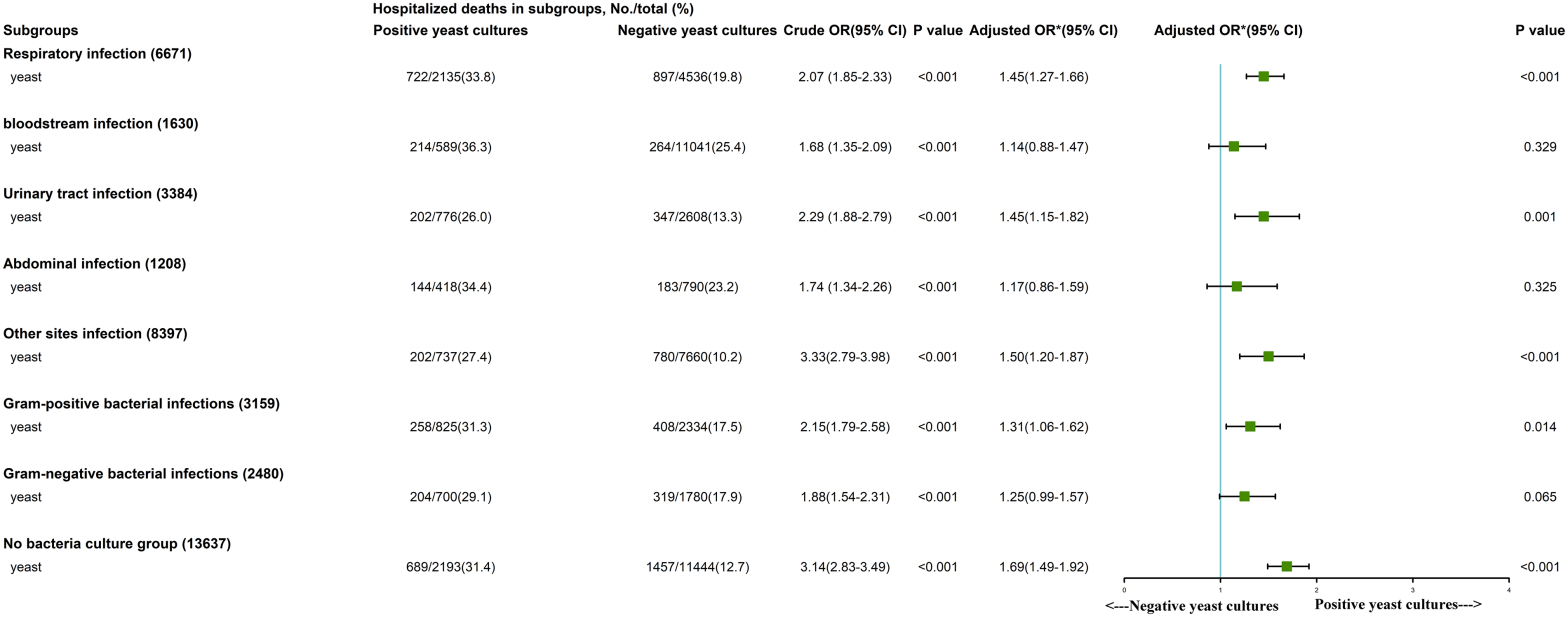
Subgroup analysis of the association between in-hospital mortality and early Positive yeast cultures. *We adjusted age, gender, ethnicity, insurance, weight, source of admission, history of disease, Sequential Organ Failure Assessment (SOFA) score, vital signs, laboratory outcomes, in-hospital management and only later antifungal agent.

Subgroup analysis showed that positive yeast culture was identified as an independent risk factor in the respiratory infection (*OR*, 1.45 [95% *CI*, 1.27–1.66]), urinary tract infection (*OR*, 1.45 [95% *CI*, 1.15–1.82]), other site infection (*OR*, 1.50 [95% *CI*, 1.20–1.87]), gram-positive bacterial infection (*OR*, 1.31 [95% *CI*, 1.06–1.62]) and bacteria-free culture groups (*OR*, 1.69 [95% *CI*, 1.49–1.92]; Figure 2). The survival curves of each subgroup were different between the two groups (Supplementary Figures S2B–I, all *p* < 0.001).

### Risk factors for in-hospital mortality among patients with early positive yeast cultures

Multivariate logistic regression analysis was performed to identify risk factors for in-hospital mortality among sepsis patients with positive yeast cultures (Table 4). Age, medications, metastatic solid tumors, severe liver disease, maximum SOFA score on the first day (*OR*, 1.09 [95% *CI*, 1.06–1.12]), maximum heart rate, maximum respiratory rate, minimum WBC count, respiratory infection, vasopressor use, early antifungal agent use (*OR*, 1.47 [95% *CI*, 1.09–1.98]), and later antifungal agent use (*OR*, 1.62 [95% *CI*, 1.28–2.03]) were independent risk factors for in-hospital mortality.

**Table 4:** Risk factors of in-hospital mortality in sepsis patients with positive yeast cultures.

Variables	Univariable			Multivariable$		
	*OR*	95% *CI*	*p*-value	*OR*	95% *CI*	*p*-value
Age	1.014	1.009–1.019	<0.001	1.019	1.013–1.026	<0.001
Male	1.028	0.891–1.187	0.702			
White	0.747	0.644–0.867	<0.001	0.718	0.608–0.848	<0.001
Insurance, Medicare	1.271	1.100–1.467	0.001	1.211	1.018–1.442	0.031
Weight	0.997	0.994–1.00	0.063	0.997	0.993–1.000	0.046
Admission (emergency)	0.803	0.695–0.927	0.003	0.975	0.831–1.145	0.760
History of disease						
Congestive heart failure	1.261	1.086–1.462	0.002	1.179	0.989–1.406	0.066
Peripheral vascular disease	1.125	0.916–1.382	0.261			
Chronic pulmonary disease	0.979	0.839–1.141	0.784			
Renal disease	1.262	0.074–1.483	0.005	0.990	0.817–1.199	0.916
Rheumatic disease	0.892	0.631–1.262	0.519			
Diabetes without cc	0.874	0.744–1.026	0.100			
Diabetes with cc	0.926	0.741–1.157	0.500			
Metastatic solid tumor	2.142	1.645–2.788	<0.001	2.447	1.762–3.398	<0.001
Severe liver disease	1.977	1.638–2.386	<0.001	1.829	1.441–2.232	<0.001
Malignant cancer	1.481	1.227–1.789	<0.001	1.126	0.888–1.428	0.327
AIDS	0.640	0.303–1.348	0.240			
Maximum SOFA score on the first day	1.125	1.106–1.145	<0.001	1.092	1.063–1.122	<0.001
Vital signs on the first day						
MAP	0.980	0.973–0.987	<0.001	0.995	0.986–1.003	0.216
Maximum heart rate	1.005	1.001–1.008	0.004	1.006	1.002–1.010	0.002
Maximum respiratory rate	1.027	1.016–1.037	<0.001	1.028	1.015–1.040	<0.001
Maximum temperature	0.726	0.666–0.792	<0.001	0.687	0.622–0.760	<0.001
Laboratory outcomes						
Minimum white blood cell	1.018	1.008–1.028	<0.001	1.049	1.027–1.071	<0.001
Maximum white blood cell	1.010	1.003–1.017	0.008	0.976	0.961–0.991	0.002
Platelets minimum	0.998	0.997–0.999	<0.001	0.999	0.998–1.000	0.015
Infection site						
Respiratory infection	1.394	1.199–1.620	<0.001	1.517	1.197–1.924	0.001
Urinary tract infection	0.728	0.609–0.871	0.001	0.727	0.590–0.897	0.003
bloodstream infection	1.328	1.103–1.599	0.003	1.215	0.975–1.512	0.082
Abdominal infection	1.188	0.958–1.475	0.117			
Central nervous infection	0.609	0.342–1.084	0.092	1.192	0.635–2.240	0.584
Other sites infection	0.798	0.666–0.956	0.014	1.140	0.846–1.537	0.388
Gram-positive bacteria	1.009	0.853–1.195	0.913			
Gram-negative bacteria	0.890	0.742–1.067	0.207			
Renal replacement therapy	1.936	1.550–2.418	<0.001	1.149	0.882–1.499	0.303
Vasopressor use	2.215	1.882–2.606	<0.001	1.445	1.168–1.787	0.001
Mechanical ventilation	1.309	1.127–1.520	<0.001	0.984	0.805–1.204	0.876
Early Antifungal agent	1.609	1.234–2.098	<0.001	1.468	1.090–1.976	0.011
Azole antifungals	0.758	0.515–1.115	0.160			
Echinocandin	3.940	2.608–5.952	<0.001			
Amphotericin	2.592	0.869–7.733	0.088			
Later antifungal agent	2.077	1.694–2.547	<0.001	1.615	1.283–2.033	<0.001

OR, odds ratio; CI, confidence interval; CC, chronic complication; SOFA, Sequential Organ Failure Assessment; AIDS, Acquired Immune Deficiency Syndrome; MAP, mean blood pressure. $First, a univariate analysis was performed. Then variables were selected for inclusion in multivariate analysis based on clinical experience and *p* < 0.1. To avoid multicollinearity issues, drug-specific variables (azole antifungals, echinocandin, amphotericin) were not included in the multivariate analysis. Hosmer-Lemeshow test for goodness of fit for multivariable logistic regression model: *χ*^2^ = 13.17, degrees of freedom = 8, *p* = 0.106.

### Effectiveness of empirical antifungal therapy among patients with positive and negative yeast cultures

**Figure 3:**
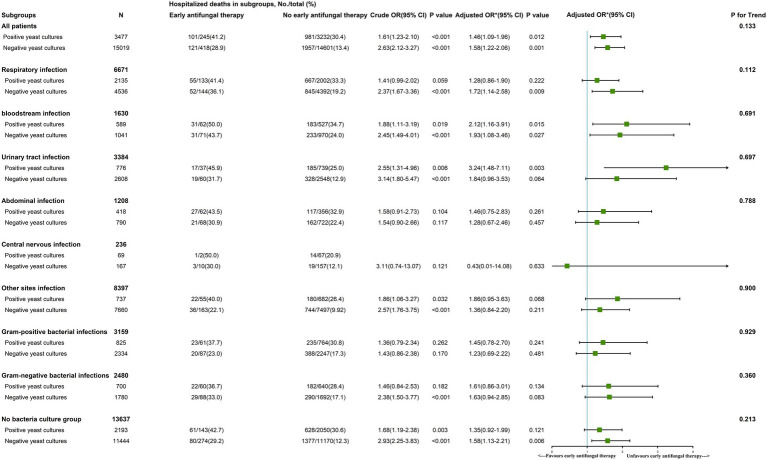
The role of early antifungal therapy in subgroups of patients with sepsis. *We adjusted age, gender, ethnicity, insurance, weight, source of admission, history of disease, SOFA score, vital signs, laboratory outcomes, in-hospital management and antifungal agents. In the last column, *p* for interaction was 0.133, indicating that there was no significant difference in the relationship between early antifungal therapy and hospital death between positive yeast cultural and negative yeast cultural. That is to say, the previous differences of 1.46 and 1.58 in *OR* were not significant, and the relationship between early antifungal therapy and hospital death could not be considered to be different in patients between positive yeast cultural and negative yeast cultural. Other parameters like respiratory infection, urinary tract infection, bloodstream infection, abdominal infection, other sites infection, gram-positive bacterial infections, gram-negative bacterial infections and no bacteria culture group are interpreted similarly to all patients groups.

Early antifungal therapy was a risk factor for in-hospital mortality in both the early positive yeast culture (*OR*, 1.46 [95% *CI*, 1.09–1.96]) and negative yeast culture groups (*OR*, 1.58 [95% *CI*, 1.22–2.06]; Figure 3).

In a subgroup analysis of the positive yeast culture group, early antifungal therapy in the bloodstream infection (*OR*, 2.12 [95% *CI*, 1.16–3.91]) and urinary tract infection groups (*OR*, 3.24 [95% *CI*, 1.48–7.11]) was a risk factor for in-hospital mortality. Early antifungal therapy did not improve outcomes in the remaining early positive yeast culture subgroups (respiratory infection, abdominal infection, other site infection, gram-positive bacterial infection, gram-negative bacterial infection, and bacteria-free culture groups; Figure 3).

In three subgroups with negative yeast cultures, namely, the respiratory infection (*OR*, 1.72 [95% *CI*, 1.14–2.58]), bloodstream infection (*OR*, 1.93 [95% *CI*, 1.08–3.46]), and bacteria-free culture groups (*OR*, 1.58 [95% *CI*, 1.13–2.21]), early antifungal treatment was a risk factor for in-hospital mortality. In the remaining subgroups with negative fungal cultures (urinary tract infection, abdominal infection, central nervous infection, other site infection, gram-positive bacterial infection, and gram-negative bacterial infection groups), early antifungal treatment did not affect in-hospital mortality (Figure 3).

## Discussion

The high detection rate of yeast has become an important clinical issue in critical care medicine, but the clinical outcomes of and empirical antifungal efficacy for patients with yeast infection are unclear. In this retrospective study, we found that patients with positive yeast cultures had higher in-hospital all-cause mortality, 60-day all-cause mortality, and longer lengths of ICU stay and hospital stay. Empirical antifungal therapy before obtaining fungal culture reports did not lower in-hospital mortality among patients with positive yeast cultures.

Evaluating the risk of fungal infection is an important issue for intensivists to consider daily. The international fungal guidelines clearly propose that the diagnosis should be based on the patient’s basic status, broad-spectrum antibiotic use, cortisol and immunosuppressant use, and relevant fungal scores. Many scores can be calculated to assess risk factors for fungal infection, including the widely used Multi-Diseases Risk Assessment Program and Candida scores (León et al., 2006). The incidence of yeast infection among critically ill patients has continued to increase (Bloos et al., 2022), and most infections are caused by *Candida albicans*, but many non-*C. albicans* fungal infections have been reported (von Lilienfeld-Toal et al., 2019). The incidence of *Candida* infection is higher among patients with a history of broad-spectrum antibiotic use, diabetes, central line catheter placement, burns, extensive surgery, especially intra-abdominal surgery, immunosuppression, renal failure, and parenteral nutrition (Sun et al., 2015; Bloos et al., 2022). This study showed that complications, disease severity and complex infections, vasopressor use, and mechanical ventilation were associated with positive yeast culture (Supplementary Table S2). This suggests that positive yeast cultures are mainly related to the invasive intubation of the natural lumen, intestinal bacterial translocation and the immune functions of the body (McGill et al., 2016; Napier et al., 2019; Sheng et al., 2021). In clinical practice, critically ill patients with the above risk factors should be monitored for possible yeast infection.

Our results revealed that patients with positive yeast cultures had higher in-hospital mortality. Subgroup analysis showed that respiratory infection, urinary tract infection, gram-positive bacterial infection and bacteria-free culture groups with positive yeast cultures had higher in-hospital all-cause mortality. The potential underlying mechanisms may be invasive intubation, dysfunction of the barrier, and administration of broad-spectrum antibiotics (McGill et al., 2016; Bassetti et al., 2019; Patel and Bergl, 2019).

Based on clinical manifestations and relevant fungal scores, intensivists often empirically administer antifungal drugs to critically ill patients with sepsis or septic shock. However, whether early antifungal therapy is effective is still largely inconclusive. Mortality rates can reach 80% among patients with candidemia if no antifungal treatment is started within the first 24 h of septic shock (Bloos et al., 2022). Studies have shown that delayed, inappropriate, or inadequate use of antifungal agents increases mortality among patients (Harbarth et al., 2003; Zaragoza et al., 2003; Garey et al., 2006). However, optimal targeted therapy is based on slow and low-sensitivity fungal culture and subsequent susceptibility testing. To start treatment as soon as possible, the European Society of Clinical Microbiology and Infectious Diseases guideline defines three untargeted treatment strategies: prophylactic therapy, preemptive therapy, and empiric therapy (Cornely et al., 2012). The clinical decision is based on risk factors, laboratory outcomes (such as β-D-glucan), and epidemiological factors. In sepsis patients with fungal infections, echinocandins are the preferred initial empiric therapy, while azoles and amphotericin B liposomes are alternatives to echinocandins (Rhodes et al., 2017). Unfortunately, a meta-analysis updated in 2016 confirmed that untargeted antifungal medicines had no effects on all-cause mortality among fungal sepsis patients (*RR* 0.93, 95% *CI*: 0.79–1.09; Cortegiani et al., 2016). A multicenter randomized clinical trial evaluated empiric antifungal therapy for suspected fungal infection and persistent fever in patients with a central catheter and demonstrated that empiric fluconazole did not improve clinical outcomes compared with placebo. Our study had similar findings: among patients with sepsis, early antifungal therapy was actually a risk factor for in-hospital mortality in many subgroups, regardless of yeast culture status. Antifungal regimens for these patients require further exploration and validation.

In general, the diagnosis and treatment of fungal sepsis is still unsatisfactory. Septic patients with positive yeast cultures had worse clinical outcomes. Although nonculture diagnostic techniques have emerged, species identification and drug susceptibility testing still rely on fungal culture. Developing rapid fungal detection methods and reducing empirical antifungal therapy use are important measures to reduce the mortality of fungal sepsis. Perhaps in the future, the use of technologies to identify yeast species, such as metagenomic next-generation sequencing tests (Li et al., 2022) or polymerase chain reaction (Kourkoumpetis et al., 2012; Donnelly et al., 2020), will be popularized. In addition, substantial heterogeneity exists in the patient population, which is a considerable challenge for personalized treatment. Individualized treatment is needed to reduce the mortality risk of patients with positive yeast cultures (Kashiha et al., 2018). The emergence of drug resistance is a potential threat to the treatment of fungal sepsis. Antifungal treatment guidelines also need to be updated according to the latest technology (Donnelly et al., 2020).

Our study is the first to determine the clinical characteristics and outcomes of patients with positive yeast cultures in a large sample. We also explored the risk factors for in-hospital mortality and studied the effect of untargeted antifungal therapies on in-hospital mortality. However, this study has some limitations. First, due to its retrospective design, the effects of confounding factors on mortality could not be thoroughly excluded. Some risk factors or test results, such as neutrophil count, were not extracted or were omitted because of too many missing values, which may have caused bias and affected the balance between the two groups. However, we used multivariate logistic regression and PSM to balance as many comorbidities and other characteristics as possible between the two groups to reduce the influence of confounding factors. Second, 95.2% (3,309/3,477) of the culture results were yeasts, and many cultured yeasts were not further classified. This may have affected the use of antifungal drugs and led to an inaccurate assessment of antifungal efficacy in this study. Third, the data were obtained from a single-center database, so the study’s conclusion should be interpreted with caution and considering actual settings. Finally, adverse effects of antifungals and readmission rates were not assessed (Whitney et al., 2019).

## Conclusion

Early yeast culture positivity increased mortality among sepsis patients, and empiric antifungal agents did not improve patient outcomes during the ICU stay prior to fungal culture reports within 48 h for patients with positive yeast cultures, and it was associated with increased in-hospital mortality among patients with negative yeast cultures. Developing rapid fungal detection methods and reducing empirical antifungal therapy use are important measures to reduce the mortality of fungal sepsis.

## Data availability statement

Publicly available datasets were analyzed in this study. This data can be found at: https://mimic.physionet.org.

## Ethics statement

This study was approved by the Research Ethics Committee of Shenzhen Second People’s Hospital (20221014001-MC). Written informed consent for participation was not required for this study in accordance with national legislation and institutional requirements.

## Author contributions

Z-yZ contributed in data management and data analysis. Z-yZ and MW conceived and designed the study. GF, J-jH, and Z-jY were responsible for literature retrieval. Z-yZ and K-jS contributed in interpretation of the data and drafting the manuscript. S-lM, FZ, and MW supervised the project and critically reviewed the final version of the paper. All authors read and approved the final manuscript, being fully accountable for ensuring its integrity and accuracy. All authors contributed to the article and approved the submitted version.

## Funding

This work were supported by grants from the Shanghai Science and Technology Innovation Action Plan (22Y11900200); the National Natural Science Foundation of China (81701899); National Key R&D Program “Stem Cell and Transformation Research” Key Special Project (2019YFA0110602); the Sanming Project of Medicine in Shenzhen (SZSM20162011), Shenzhen Science and Technology Innovation Commission (JCYJ20190806163603504), Shenzhen Second People’s Hospital Clinical Research Fund of Guangdong Province High-level Hospital Construction Projects (20173357201815, 20193357003, and 20203357014).

## Conflict of interest

The authors declare that the research was conducted in the absence of any commercial or financial relationships that could be construed as a potential conflict of interest.

## Publisher’s note

All claims expressed in this article are solely those of the authors and do not necessarily represent those of their affiliated organizations, or those of the publisher, the editors and the reviewers. Any product that may be evaluated in this article, or claim that may be made by its manufacturer, is not guaranteed or endorsed by the publisher.
