# Supplementary material for: Impact of early empirical antifungal therapy on prognosis of sepsis patients with positive yeast culture: A retrospective study from the MIMIC-IV database
Source: Front Microbiol. 2022 Nov 17;13:1047889. doi: 10.3389/fmicb.2022.1047889 (PMC9712452; doi:10.3389/fmicb.2022.1047889)
Supplement: Supplementary file 1 [file Data_Sheet_1.ZIP › Supplementary materials/Fig S1.docx]

Figure S1. Standardized mean difference (SMD) of variables before and after PSM.


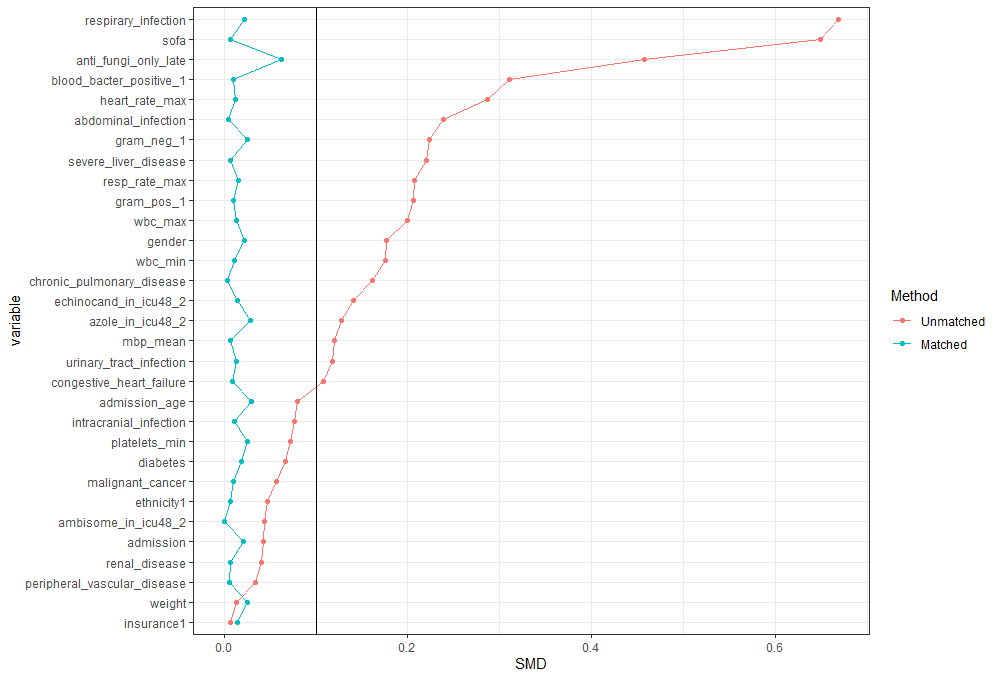


Abbreviation: SMD standardized mean difference; CC chronic complication；SOFA Sequential Organ Failure Assessment; MAP mean blood pressure.
