# Supplementary material for: Impact of early empirical antifungal therapy on prognosis of sepsis patients with positive yeast culture: A retrospective study from the MIMIC-IV database
Source: Front Microbiol. 2022 Nov 17;13:1047889. doi: 10.3389/fmicb.2022.1047889 (PMC9712452; doi:10.3389/fmicb.2022.1047889)
Supplement: Supplementary file 1 [file Data_Sheet_1.ZIP › Supplementary materials/Table S1.docx]

**Table S1. Missing number (%) for risk variables and outcome variables**

| Risk variables^$^ | Missing number (%) |
| --- | --- |
| Age | 0(0) |
| Male | 0(0) |
| White | 0(0) |
| Insurance, Medicare | 0(0) |
| Weight | 224(1.21) |
| Admission（emergency） | 0(0) |
| History of disease | 0(0) |
| Congestive heart failure | 0(0) |
| Peripheral vascular disease | 0(0) |
| Chronic pulmonary disease | 0(0) |
| Renal disease | 0(0) |
| Rheumatic disease | 0(0) |
| Diabetes without cc | 0(0) |
| Diabetes with cc | 0(0) |
| Metastatic solid tumor | 0(0) |
| Severe liver disease | 0(0) |
| Malignant cancer | 0(0) |
| Aids | 0(0) |
| Maximum SOFA score on the first day | 0(0) |
| Vital signs on the first day | 0(0) |
| Mean MAP | 17(0.09) |
| Maximum heart rate | 16(0.09) |
| Maximum respiratory rate | 27(0.12) |
| Maximum temperature | 995(5.38) |
| Laboratory outcomes | 0(0) |
| Minimum white blood cell | 53(0.29) |
| Maximum white blood cell | 53(0.29) |
| Platelets min | 55(0.30) |
| Infection site | 0(0) |
| Respiratory infection | 0(0) |
| Urinary tract infection | 0(0) |
| bloodstream infection | 0(0) |
| Abdominal infection | 0(0) |
| Central nervous infection | 0(0) |
| Other sites infection | 0(0) |
| Gram-positive bacteria | 0(0) |
| Gram-negative bacteria | 0(0) |
| Renal replacement therapy | 0(0) |
| Vasopressor-use | 0(0) |
| Mechanical ventilation | 0(0) |
| Early Antifungal agent | 0(0) |
| Azole antifungals | 0(0) |
| Echinocandin | 0(0) |
| Amphotericin | 0(0) |
| Later antifungal agent | 0(0) |
| Primary outcome | 0(0) |
| In-hospital mortality | 0(0) |
| Secondary outcomes | 0(0) |
| 30-day mortality | 0(0) |
| 60-day mortality | 0(0) |
| Length of ICU stay | 0(0) |
| Length of hospital stay | 0(0) |

Abbreviation: CC chronic complication； SOFA Sequential Organ Failure Assessment; MAP mean blood pressure.

^$^The proportion of missing values is less than 6%. Multiple imputations were used for missing values under the assumption of missing at random.
