# Supplementary material for: Impact of early empirical antifungal therapy on prognosis of sepsis patients with positive yeast culture: A retrospective study from the MIMIC-IV database
Source: Front Microbiol. 2022 Nov 17;13:1047889. doi: 10.3389/fmicb.2022.1047889 (PMC9712452; doi:10.3389/fmicb.2022.1047889)
Supplement: Supplementary file 1 [file Data_Sheet_1.ZIP › Supplementary materials/Table S2.docx]

**Table S2. risk factors associated with positive yeast cultures**

|  | Univariable |  |  | Multivariable^$^ |  |  |
| --- | --- | --- | --- | --- | --- | --- |
| Variables | OR | 95% CI | P value | OR | 95% CI | P value |
| Age | 0.995 | 0.993-0.997 | <0.001 | 0.995 | 0.992-0.998 | 0.001 |
| Male | 0.694 | 0.644-0.747 | <0.001 | 0.621 | 0.570-0.677 | <0.001 |
| White | 0.915 | 0.847-0.988 | 0.024 | 1.059 | 0.969-1.158 | 0.203 |
| Insurance, Medicare | 1.019 | 0.946-1.097 | 0.624 |  |  |  |
| Weight | 1.001 | 0.999-1.002 | 0.378 |  |  |  |
| Admission（emergency） | 1.104 | 1.025-1.188 | 0.009 | 1.089 | 1.000-1.186 | 0.049 |
| History of disease |  |  |  |  |  |  |
| Congestive heart failure | 1.237 | 1.145-1.337 | <0.001 | 1.122 | 1.019-1.235 | 0.019 |
| Peripheral vascular disease | 1.099 | 0.987-1.224 | 0.086 | 1.192 | 1.053-1.350 | 0.006 |
| Chronic pulmonary disease | 1.413 | 1.304-1.531 | <0.001 | 1.280 | 1.167-1.404 | <0.001 |
| Renal disease | 1.094 | 1.005-1.190 | 0.038 | 0.962 | 0.861-1.075 | 0.497 |
| Rheumatic disease | 1.265 | 1.059-1.512 | 0.010 | 1.156 | 0.944-1.415 | 0.160 |
| Diabetes without cc | 1.183 | 1.089-1.284 | <0.001 | 1.203 | 1.096-1.321 | <0.001 |
| Diabetes with cc | 1.110 | 0.991-1.244 | 0.072 | 1.218 | 1.060-1.398 | 0.005 |
| Metastatic solid tumor | 1.077 | 0.931-1.247 | 0.317 |  |  |  |
| Severe liver disease | 1.979 | 1.776-2.206 | <0.001 | 1.654 | 1.435-1.906 | <0.001 |
| Malignant cancer | 1.162 | 1.049-1.286 | 0.004 | 1.163 | 1.034-1.308 | 0.012 |
| Aids | 1.445 | 1.008-2.071 | 0.045 | 1.522 | 1.006-2.304 | 0.047 |
| Maximum SOFA score on the first day | 1.167 | 1.156-1.177 | <0.001 | 1.120 | 1.104-1.137 | <0.001 |
| Vital signs on the first day |  |  |  |  |  |  |
| Mean MAP | 0.987 | 0.984-0.991 | <0.001 | 0.997 | 0.993-1.001 | 0.180 |
| Maximum heart rate | 1.013 | 1.011-1.015 | <0.001 | 1.005 | 1.003-1.007 | <0.001 |
| Maximum respiratory rate | 1.030 | 1.025-1.036 | <0.001 | 1.002 | 0.996-1.009 | 0.478 |
| Maximum temperature | 1.104 | 1.054-1.155 | <0.001 | 0.945 | 0.896-0.997 | 0.037 |
| Laboratory outcomes |  |  |  |  |  |  |
| Minimum white blood cell | 1.028 | 1.023-1.034 | <0.001 | 1.023 | 1.011-1.035 | <0.001 |
| Maximum white blood cell | 1.022 | 1.018-1.026 | <0.001 | 0.988 | 0.980-0.997 | 0.006 |
| Platelets min | 1.001 | 1.000-1.001 | <0.001 | 1.002 | 1.002-1.002 | <0.001 |
| Infection sites |  |  |  |  |  |  |
| Respiratory infection | 3.677 | 3.405-3.970 | <0.001 | 2.956 | 2.596-3.368 | <0.001 |
| Urinary tract infection | 1.367 | 1.249-1.496 | <0.001 | 1.247 | 1.108-1.402 | <0.001 |
| Bloodstream infection | 2.738 | 2.456-3.053 | <0.001 | 1.661 | 1.447-1.907 | <0.001 |
| Abdominal infection | 2.461 | 2.172-2.788 | <0.001 | 1.636 | 1.398-1.915 | <0.001 |
| Central nervous infection | 1.801 | 1.357-2.389 | <0.001 | 2.179 | 1.578-3.009 | <0.001 |
| Other sites infection | 0.258 | 0.237-0.282 | <0.001 | 0.933 | 0.794-1.095 | 0.394 |
| Gram-positive bacteria | 1.691 | 1.546-1.849 | <0.001 | 1.194 | 1.076-1.326 | 0.001 |
| Gram-negative bacteria | 1.875 | 1.702-2.065 | <0.001 | 1.316 | 1.177-1.470 | <0.001 |
| Renal replacement therapy | 1.698 | 1.495-1.930 | <0.001 | 0.806 | 0.685-0.949 | 0.009 |
| Vasopressor-use | 2.042 | 1.892-2.205 | <0.001 | 1.160 | 1.043-1.289 | 0.006 |
| Mechanical ventilation | 2.047 | 1.898-2.207 | <0.001 | 1.284 | 1.159-1.421 | <0.001 |
| Early Antifungal agent | 2.648 | 2.251-3.11 | <0.001 | 1.568 | 1.295-1.899 | <0.001 |
| Azole antifungals | 2.258 | 1.835-2.777 | <0.001 |  |  |  |
| Echinocandin | 2.975 | 2.302-3.845 | <0.001 |  |  |  |
| Amphotericin | 2.345 | 1.193-4.610 | 0.013 |  |  |  |
| Later antifungal agent | 9.740 | 8.243-11.508 | <0.001 | 5.187 | 4.300-6.257 | <0.001 |

Abbreviation: OR odds ratio; CI confidence interval; CC chronic complication； SOFA Sequential Organ Failure Assessment; MAP mean blood pressure.

$ First, a univariate analysis was performed. Then variables were selected for inclusion in multivariate analysis based on clinical experience and P value less than 0.1.

To avoid multicollinearity issues, drug-specific variables (Azole antifungals, Echinocandin, Amphotericin) were not included in the multivariate analysis.
