# Supplementary figures and images for: Impact of early empirical antifungal therapy on prognosis of sepsis patients with positive yeast culture: A retrospective study from the MIMIC-IV database
Source: Front Microbiol. 2022 Nov 17;13:1047889. doi: 10.3389/fmicb.2022.1047889 (PMC9712452; doi:10.3389/fmicb.2022.1047889)

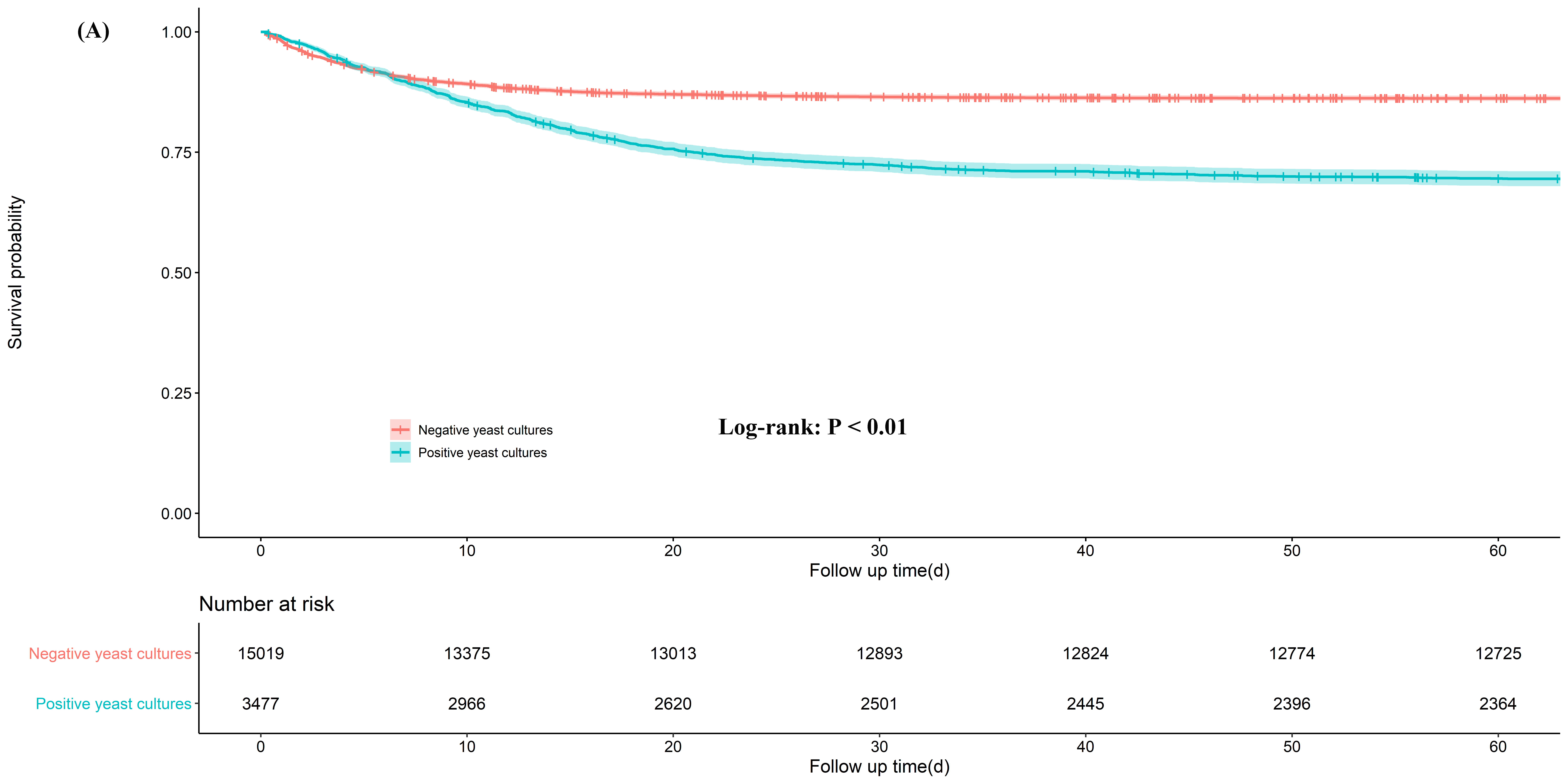

Supplement: Supplementary file 1 [file Data_Sheet_1.ZIP › Supplementary materials/Fig S2(A).tif]

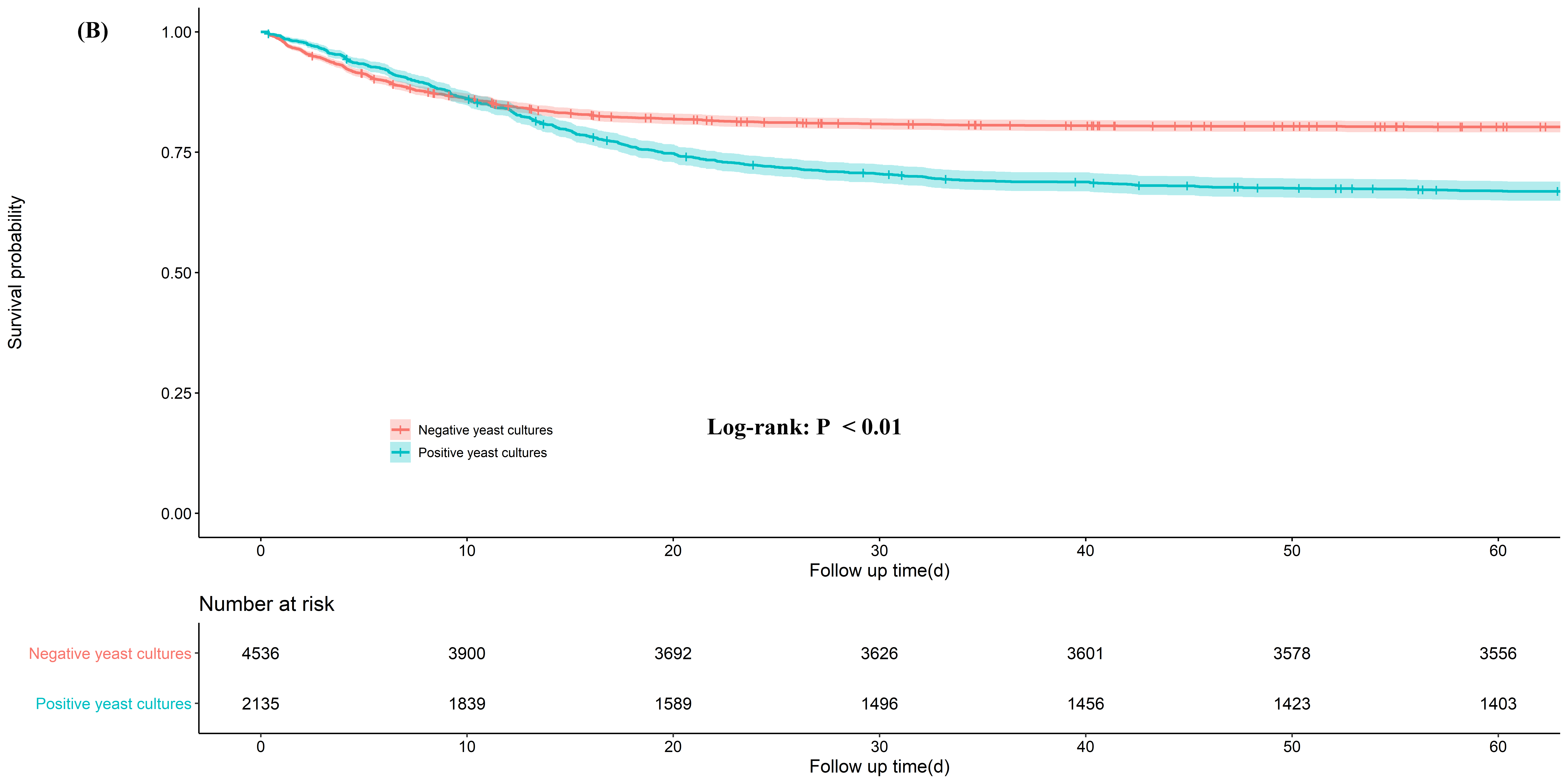

Supplement: Supplementary file 1 [file Data_Sheet_1.ZIP › Supplementary materials/Fig S2(B).tif]

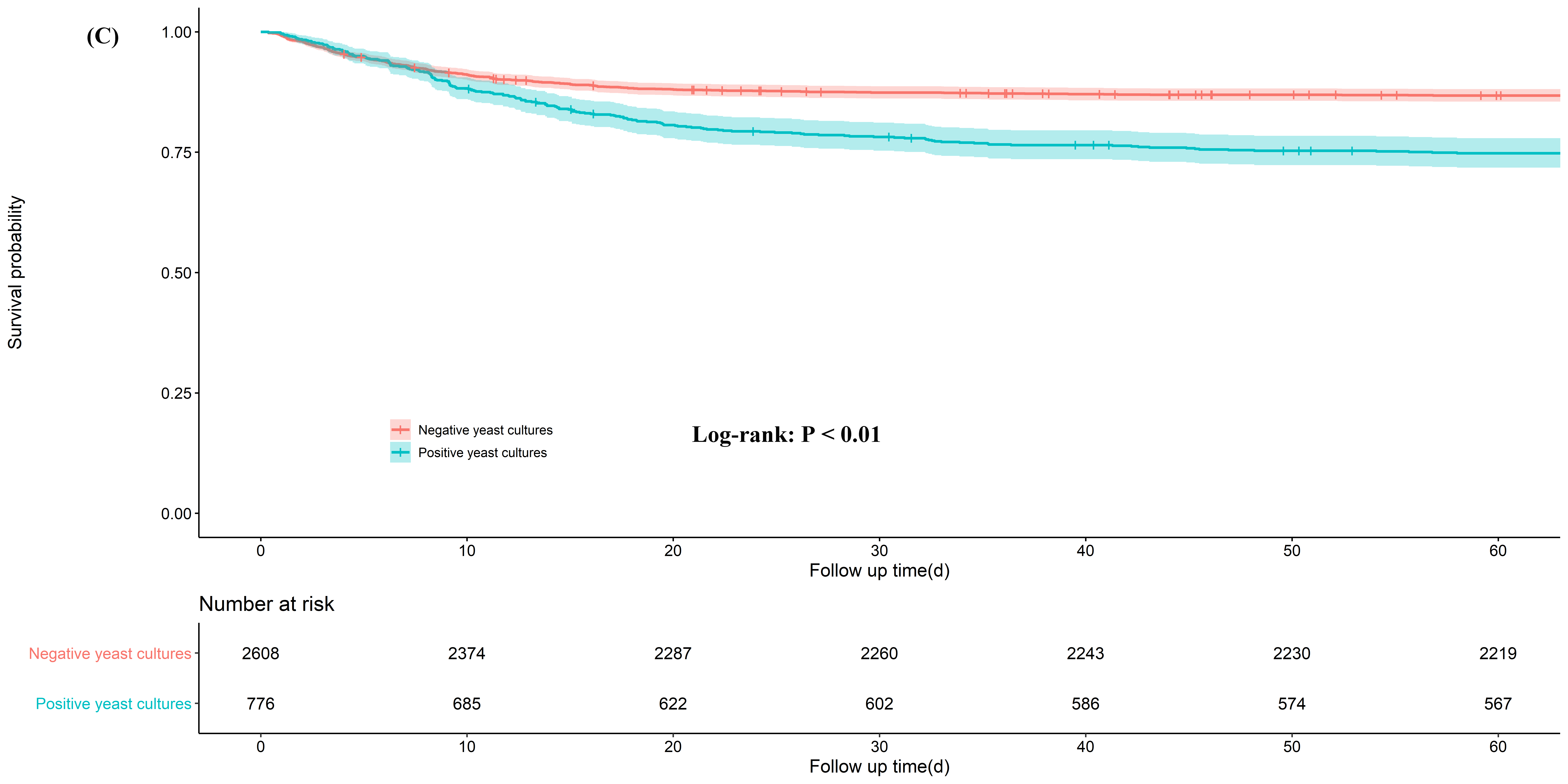

Supplement: Supplementary file 1 [file Data_Sheet_1.ZIP › Supplementary materials/Fig S2(C).tif]

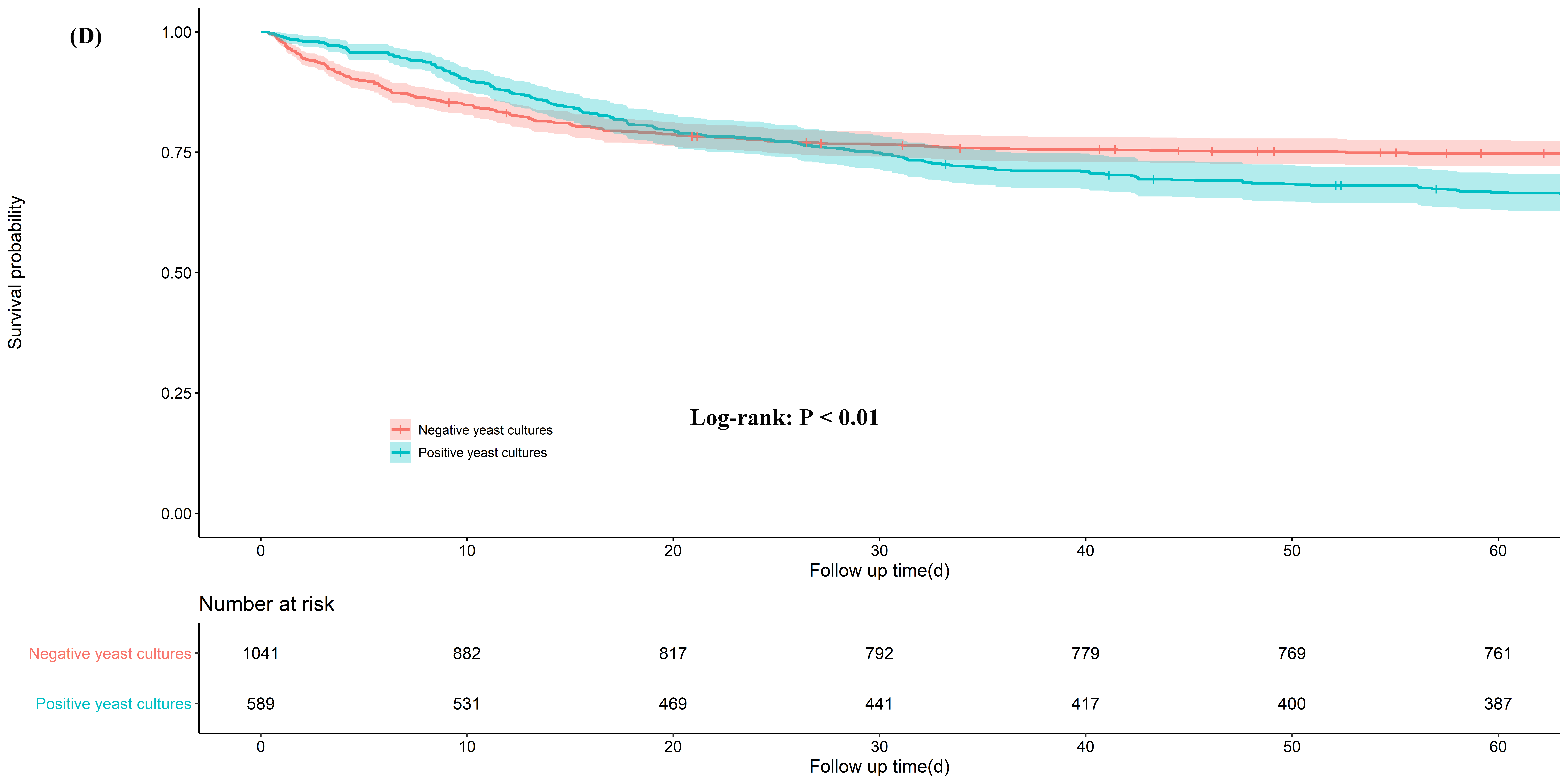

Supplement: Supplementary file 1 [file Data_Sheet_1.ZIP › Supplementary materials/Fig S2(D).tif]

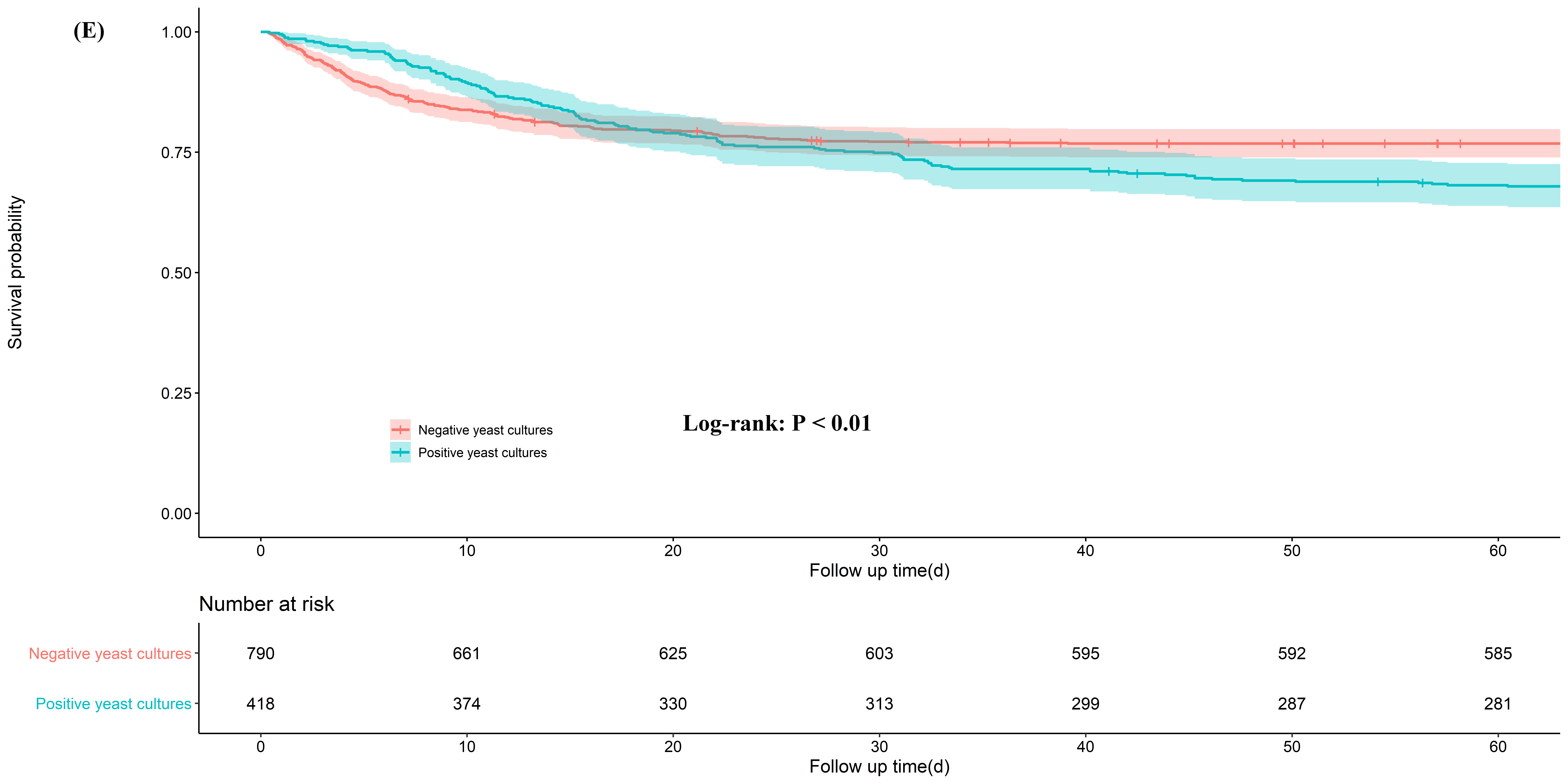

Supplement: Supplementary file 1 [file Data_Sheet_1.ZIP › Supplementary materials/Fig S2(E).tif]

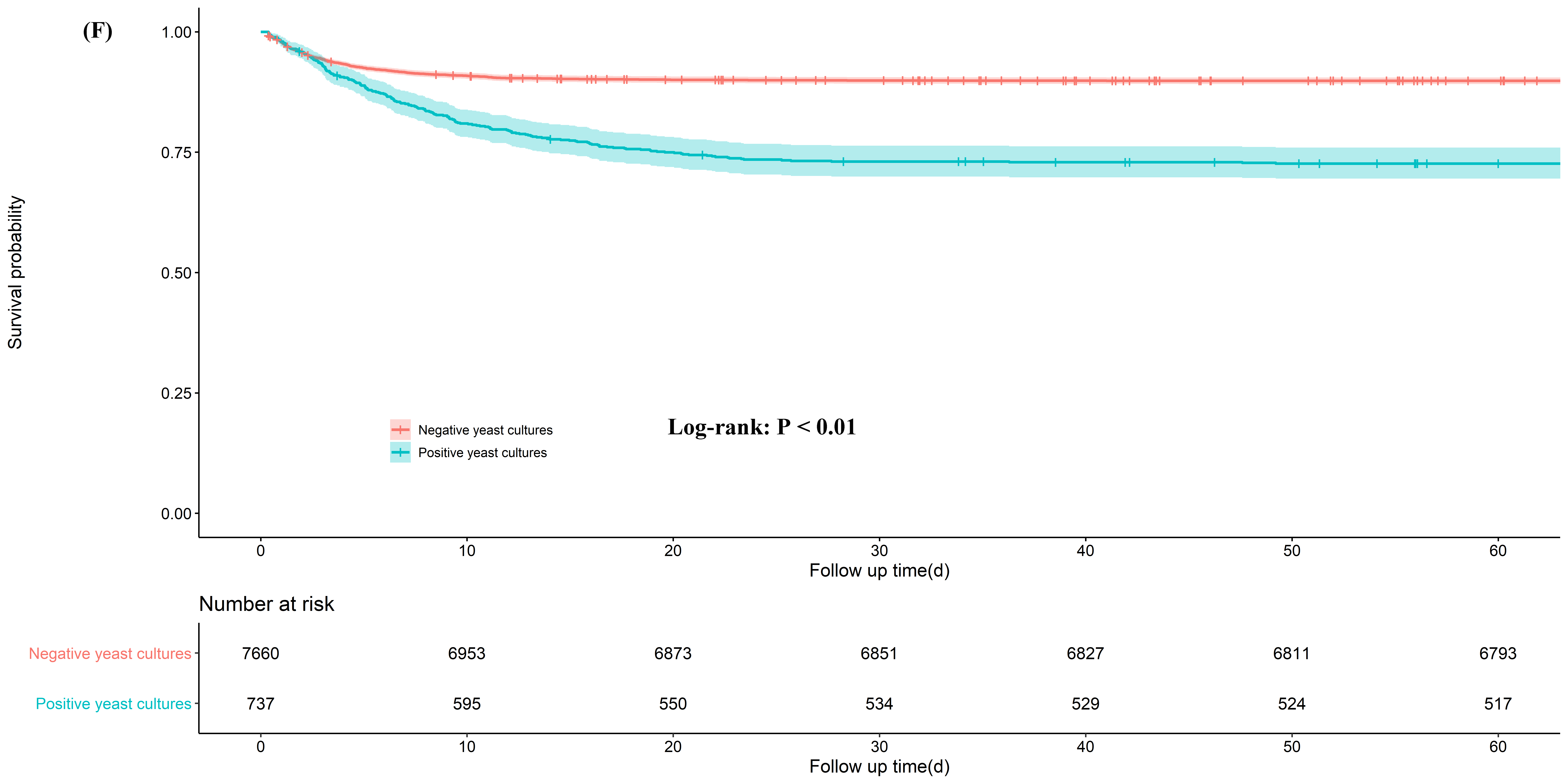

Supplement: Supplementary file 1 [file Data_Sheet_1.ZIP › Supplementary materials/Fig S2(F).tif]

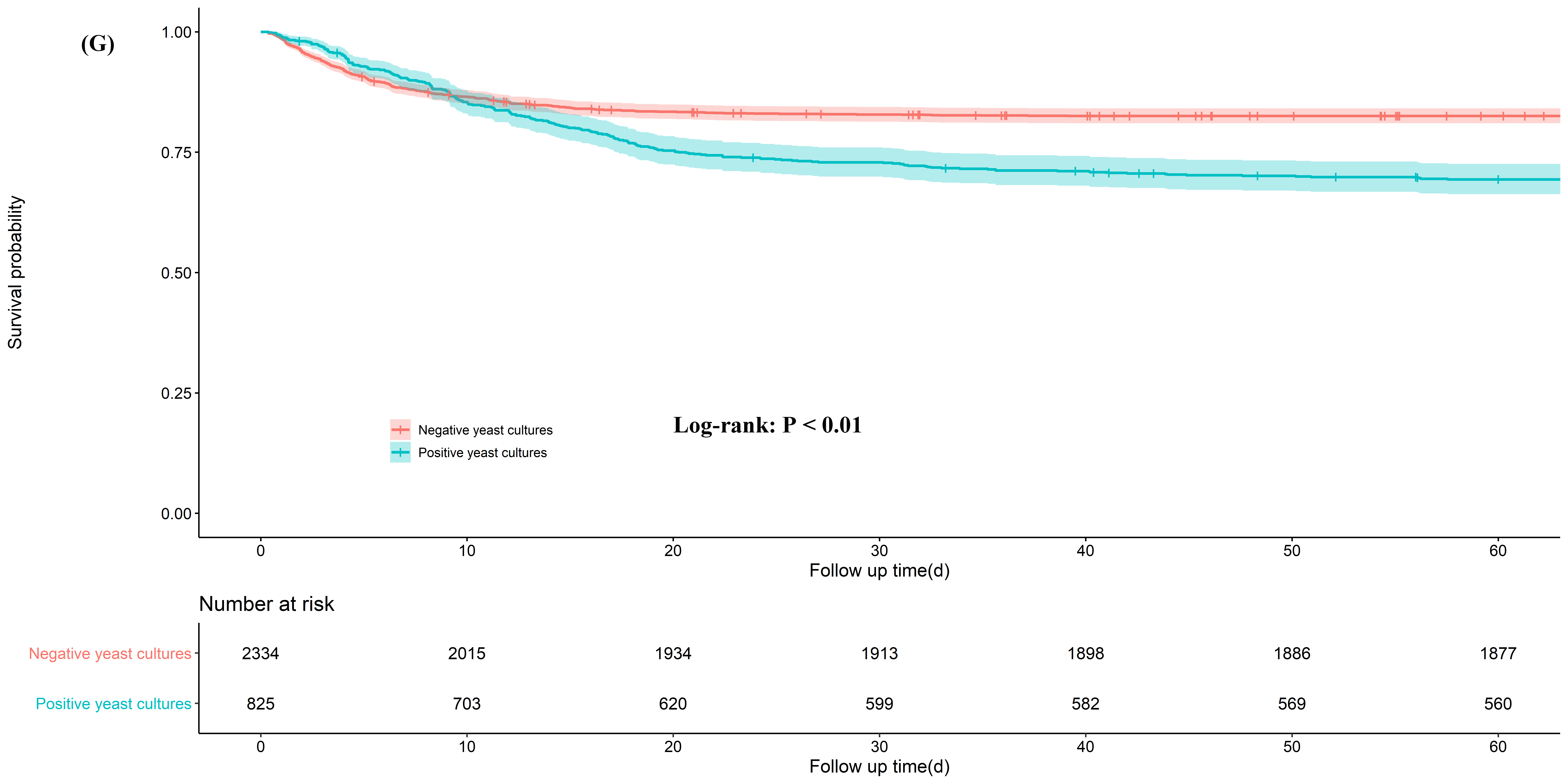

Supplement: Supplementary file 1 [file Data_Sheet_1.ZIP › Supplementary materials/Fig S2(G).tif]

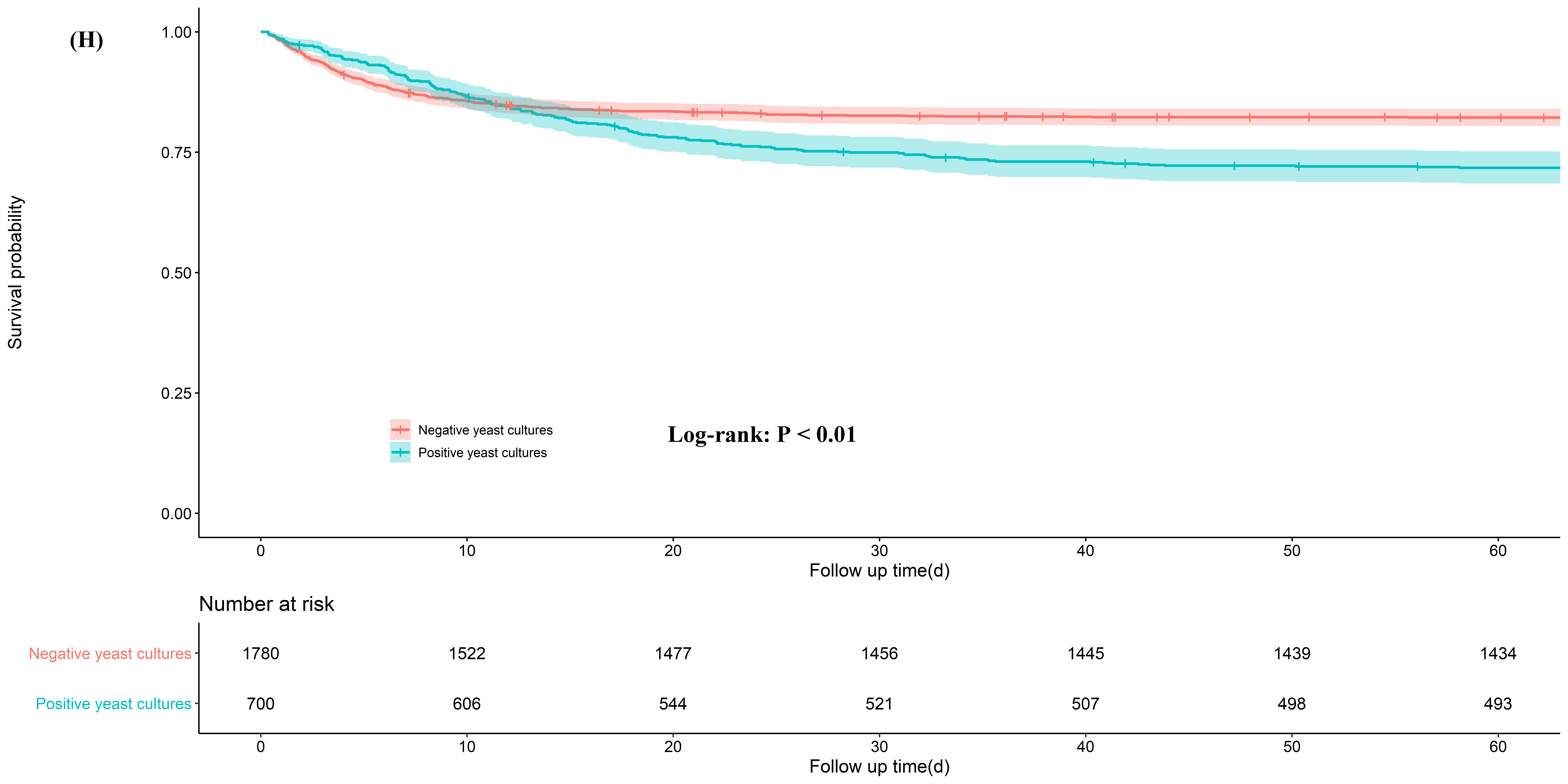

Supplement: Supplementary file 1 [file Data_Sheet_1.ZIP › Supplementary materials/Fig S2(H).tif]

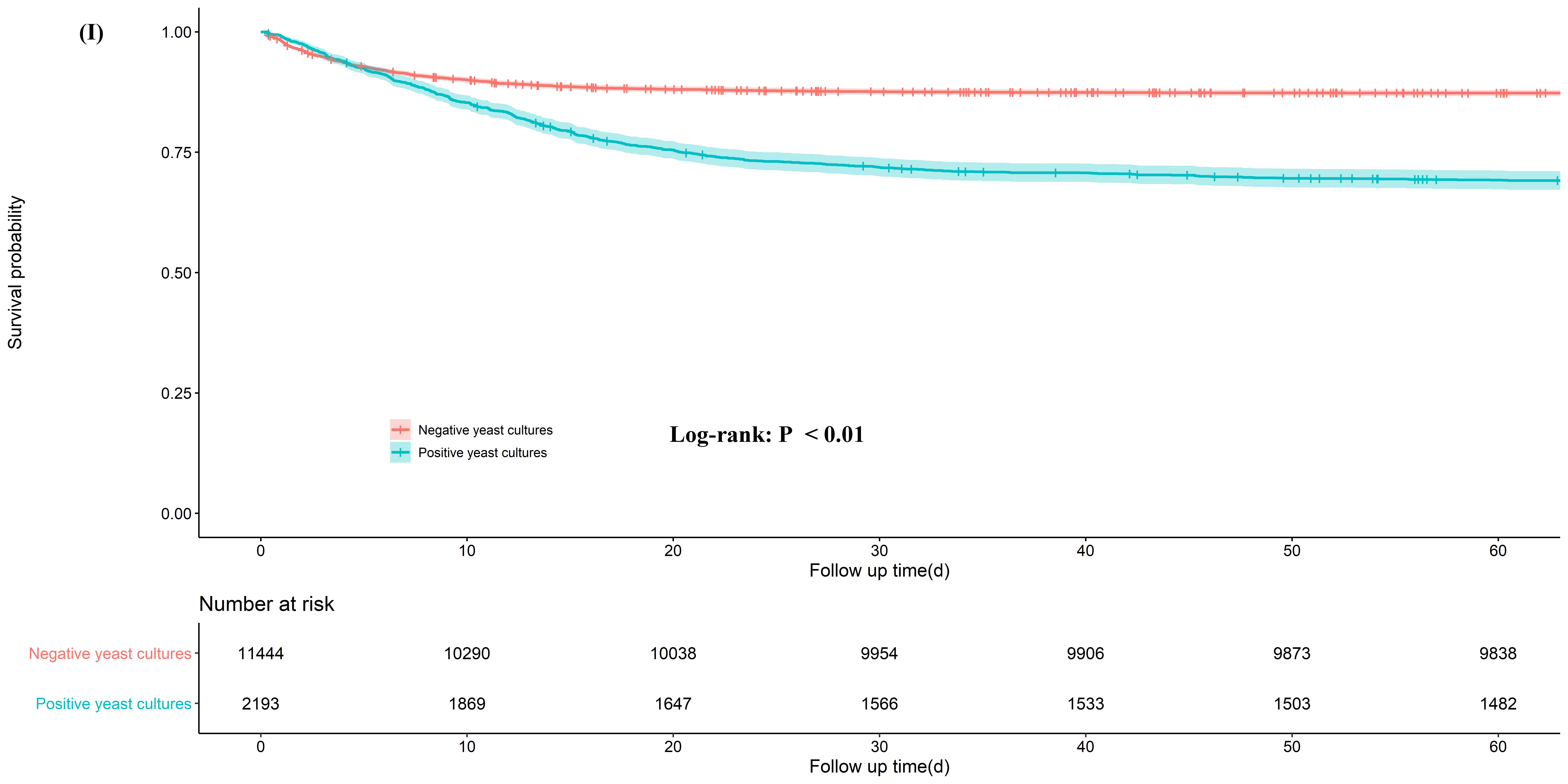

Supplement: Supplementary file 1 [file Data_Sheet_1.ZIP › Supplementary materials/Fig S2(I).tif]
