# Supplementary material for: Impact of early empirical antifungal therapy on prognosis of sepsis patients with positive yeast culture: A retrospective study from the MIMIC-IV database
Source: Front Microbiol. 2022 Nov 17;13:1047889. doi: 10.3389/fmicb.2022.1047889 (PMC9712452; doi:10.3389/fmicb.2022.1047889)
Supplement: Supplementary file 1 [file Data_Sheet_1.ZIP › Supplementary materials/Table S3.doc]

**Table S3****. Infection diagnosis, species, and in-hospital mortality of fungi infections**

| Type of culture | Other yeast fungi, n | Candida spp., n | Fungi-positive patients* (N) | Hospital mortality, n (%) |
| --- | --- | --- | --- | --- |
| Respiratory infection | 2084 | 234 | 2135 | 722(33.8) |
| Urinary tract infection | 760 | 74 | 776 | 202(26.0) |
| bloodstream infection | 544 | 153 | 589 | 214(36.3) |
| Abdominal infection | 381 | 108 | 418 | 144(34.4) |
| Central nervous infection | 66 | 8 | 69 | 15(21.7) |
| Other sites infection | 674 | 109 | 737 | 202(27.4) |

*Some patients have both Candida and yeast culture results.
